# Methylene tetrahydrofolate reductase gene mutation in sickle cell anaemia patients in Lagos, Nigeria

**DOI:** 10.11604/pamj.2019.34.213.19524

**Published:** 2019-12-30

**Authors:** Oluwaseun Olabisi Adelekan, Ebele Ifeyinwa Uche, Taiwo Modupe Balogun, Vincent Oluseye Osunkalu, Akinsegun Abduljaleel Akinbami, Kamal Ayobami Ismail, Mulikat Adesola Badiru, Adedoyin Owolabi Dosunmu, Omolara Risqat Kamson

**Affiliations:** 1 Department of Haematology and Blood Transfusion, General Hospital Marina, Lagos, Nigeria; 2 Department of Haematology and Blood Transfusion, Lagos State University College of Medicine, Lagos, Nigeria; 3 Department of Haematology and Blood Transfusion, Lagos State University Teaching Hospital, Lagos, Nigeria; 4 Department of Haematology and Blood Transfusion, College of Medicine, University of Lagos, Idiaraba, Lagos, Nigeria

**Keywords:** Sickle cell anaemia, methylene tetrahydrofolate reductase gene mutation, C677T, A1298C

## Abstract

**Introduction:**

The significant causes of mortality among individuals with sickle cell anaemia (SCA) such as acute chest syndrome and cerebrovascular disease are related to vascular occlusion. Polymorphisms of the methylene tetrahydrofolate reductase (MTHFR) gene in persons with sickle cell anaemia have been suggested as a potential risk for vaso-occlusive events, with the C677T and A1298C polymorphisms being the commonest. This study therefore aimed to establish the pattern of MTHFR C677T and A1298C gene mutations among adults with HbSS phenotype attending the Haematology Clinic in Lagos State University Teaching Hospital Lagos, Nigeria.

**Methods:**

A cross-sectional study was done among SCA patients attending the Haematology Clinic of the Lagos State University Teaching Hospital (LASUTH), using age and sex matched HbAA controls. DNA extraction and gene analysis were done. The selective amplification of a particular segment of the DNA by polymerase chain reaction (PCR) was done and subsequent digestion of the amplified MTHFR gene into its various fragments.

**Results:**

The overall prevalence of the C677T mutation among participants was 19.3% (37 of 192), while the prevalence of A1298C was 15% (29 of 192).

**Conclusion:**

The prevalence of MTHFR C677T was higher than A1298C mutations among sickle cell anaemia subjects.

## Introduction

Sickle cell anaemia has been recognized as a problem of major public health significance by the World Health Organization [1]. The highest frequency of the disease is found in tropical regions particularly sub-Saharan Africa, India and the Middle East. More than 75% of sickle cell anaemia cases occur in sub-Saharan Africa [1]. Nigeria has the highest burden of sickle cell anaemia worldwide, with prevalence values ranging from 2% to 3% of the 140 million population [2-5]. Haemolytic and vaso-occlusive complications are seen in patients with sickle cell anaemia as well as a heightened occurrence of thrombo-embolic complications [6-9]. Thrombo-embolic complications remain a significant cause of morbidity and mortality in people with sickle cell anaemia [6, 10, 11]. These complications include stroke [12, 13], avascular necrosis of the head of femur [14], thrombotic microangiopathy [15], venous thromboembolism [16], retinopathy [17], end organ damage [18, 19], chronic leg ulcers [18, 19] amongst others. Hypercoagulability in sickle cell anaemia may be responsible for the increased development of vascular occlusion in certain organs [6, 19] and acute pain episodes [6, 20]. The causes of hypercoagulability in sickle cell anaemia are multifactorial. Factors such as increased plasma levels of homocysteine, reduced levels of natural anticoagulants like proteins C and S [21], increased levels of thrombin generation markers like thrombin-antithrombin (TAT) complexes or protein fragment 1+2 (F1+2) [22], increased D-dimer complexes, circulating antiphospholipid antibodies, increased tissue factor expression and adherence of sickled erythrocytes to the vascular endothelium, Factor V Leiden and prothrombin gene mutation have all been implicated [7, 8]. The MTHFR gene is 2.2 kilobases long and located on chromosome 1 at 1p36.3 [23, 24]. The complimentary DNA sequence is 2.2 kilobases long and consists of 11 exons [25]. This gene codes for the protein methylenetetrahydrofolate reductase (MTHFR) which catalyzes the conversion of 5, 10-methylenetetrahydrofolate to 5-methyltetrahydrofolate [23, 24]. 5-methyltetrahydrofolate is a major form of folate in the plasma [23, 24, 26] and it is a co-factor in the re-methylation of homocysteine to methionine thereby reducing plasma homocysteine levels [23, 24, 26]. Folate in its 5-methyl form, also participates in single-carbon transfers which occur as part of the synthesis of nucleotides and DNA methylation [24]. It is also involved in the synthesis of proteins, neurotransmitters and phospholipids [24]. Normal MTHFR activity maintains the level of circulating folate and methionine and possibly prevents accumulation of homocysteine [24]. Therefore, mutations in the MTHFR gene reduces the activity of the enzyme MTHFR leading to hyper-homocysteinaemia [23, 24, 26] and this is particularly heightened in patients with sickle cell anaemia who have an increased demand for folate due to their high red cell turnover or shortened red cell lifespan.

C677T and A1298C are the two most commonly studied polymorphisms of the MTHFR gene, which are associated with diminished enzyme activity of methylene tetrahydrofolate reductases resulting in the accumulation of homocysteine especially in individuals with low levels of folate [24, 27-30]. The significant causes of mortality among individuals with sickle cell anaemia such as acute chest syndrome and cerebrovascular disease are related to vascular occlusion [12, 31-36]. Polymorphisms of the MTHFR gene in persons with sickle cell anaemia have been suggested as a potential risk for vaso-occlusive events [37-41]. Furthermore, avascular necrosis of the femoral and humeral head is a common musculoskeletal complication seen in adults with sickle cell anaemia which reduces their quality of life [42]. MTHFR mutations have also been implicated as a probable risk factor for avascular necrosis in sickle cell anaemia [43-45]. This study aimed to establish the pattern of MTHFR gene mutations among adults with HbSS phenotype attending the Haematology Clinic of the Lagos State University Teaching Hospital (LASUTH) with a view to determine the role of MTHFR gene polymorphism as a contributory factor in their differential phenotypic expression. Also, very few studies have been published involving MTHFR gene mutations from Africa, therefore, our study would add to the body of knowledge currently available on the MTHFR gene mutations.

## Methods

### Study area and population

The study was conducted among adult patients with sickle cell anaemia attending the Haematology Clinic of LASUTH, Lagos, Nigeria, while apparently healthy individuals with no known medical condition served as controls. LASUTH is a major tertiary healthcare facility, located in the south-western part of Nigeria, within the Lagos metropolis.

### Study design

This was a cross-sectional study.

### Study period

The study was carried out over a period of eight months from May 2016 to December 2016.

### Sampling technique

Purposive non-probability sampling technique was used to recruit presumably healthy staff and students of LASUTH Ikeja with haemoglobin phenotype AA and they served as the control group. A sampling frame containing the list of individuals with sickle cell anaemia attending the adult Haematology Clinic in LASUTH was drawn and subjects were selected randomly. However, only individuals with a haemoglobin electrophoresis result showing the SS band were enrolled into the study. The inclusion criteria were: age between 18 and above; known patients with sickle cell anaemia (screened by the haemoglobin S solubility test and diagnosed by cellulose acetate electrophoresis at pH 8.6) who are in steady state defined as the period free of crisis extending from at least three weeks since the last clinical event and three months or more since the last blood transfusion, to at least one week before the start of a new clinical event [46]; haemoglobin AA persons (confirmed by cellulose acetate electrophoresis at pH 8.6) who served as the control group; those who met the above criteria and agreed to participate in the study by signing the informed consent form. The participants were classified as follows: adults with sickle cell anaemia in steady state; HbAA controls who were age and sex-matched with the adults with sickle cell anaemia. The exclusion criteria were: confirmed phenotype HbAC, HbAS; history of acute or chronic illness e.g. any febrile illness, hypertension, diabetes, epilepsy, asthma; intravenous drug users; non consenting participants.

### Sample size determination

Sample size was determined using the formula for prevalence study by Daniel [47, 48].

$$n=\frac{Z^{2}pq}{d^{2}}$$

Where: n = sample size; Z = statistic for a level of confidence of 95%, which is conventional, Z value is 1.96; p = expected prevalence; q = 1-p; d = precision. Therefore, considering Z = 1.96; p = prevalence of MTHFR gene mutation among sub-Saharan Africans is 6% [49, 50]; p = 0.06; q = 1-p; d = 0.05; n = (1.96)^2^ x 0.06 x (1-0.06) / (0.05)^2^; n = 3.8416 x 0.06 x 0.94 / 0.0025; n = 0.216667 / 0.0025; n = 86.67; n = 87 (which is the minimum sample size in each group).

However, in order to accommodate possible attrition or unforeseen errors in completing the study questionnaire or blood sample processing, an additional 10% (9 patients) of the calculated figure was added. Therefore, a total of 192 participants (96 in each group) were recruited into this study.

### Ethical consideration and clearance

Ethical approval was obtained from the Health Research and Ethics Committee of LASUTH prior to commencement of the study.

### Participants' informed consent

A written informed consent was obtained from all the participants before being recruited into the study. No participant was in any way coerced or cajoled to participate in this study. Study was done at no cost to the participants.

### Confidentiality

The names and initials of all participants were not recorded or used to guarantee confidentiality. Participants were instead given code numbers. Paper records were secured in a cabinet inside a secured room. Electronic data was protected using a password known only to the researchers.

### Questionnaire administration and history taking

Each participant was interviewed to obtain relevant demographic and clinical data with the use of a questionnaire. The questionnaire was administered to each participant by a member of the research team. Information on the HbSS subjects and their medical history including disease complications were retrieved from their case notes.

### Specimen collection and storage

Seven milliliters of venous blood was drawn from each subject. Five milliliters of this was dispensed into sodium ethylene diamine tetra-acetate (EDTA) specimen bottles. This sample was used for a full blood count (FBC). This was analyzed within 2 hours of collection. Some of the blood collected in the EDTA specimen bottle was used for cellulose acetate electrophoresis test. The remaining two milliliters of the blood was dispensed into another set of EDTA bottles. These samples were prepared for DNA extraction and subsequently used for genetic analysis. The samples were washed with phosphate buffer saline (PBS) until the supernatant was white. The samples were then stored at the appropriate temperature till the DNA extraction was done. It was stored at 2-8°C if the DNA extraction was scheduled to be done within a week, at -20°C within a month and -80°C for a longer period of time.

### Statistical analysis

All data obtained were entered into and analyzed using Statistical Package for Social Sciences (SPSS), version 22.0, Chicago, USA. Data summaries were presented in simple tables and frequencies. Data obtained from the study were evaluated for normality using the Kolmogorov-Smirnov test. Numerical data were expressed as mean±SD or median values for parametric and non-parametric data respectively. The differences in mean distribution of variables were tested using student t-test or ANOVA where appropriate (significant level was set at p < 0.05 or F < 0.05 for student t-test and ANOVA respectively). The degree of association between variables were evaluated with chi-square test and significance was set at p < 0.05.

## Results

### Age and gender

This was a cross-sectional study of 192 participants consisting of 96 SCA patients (cases) and 96 HbAA genotype (controls). A total of 202 participants were initially recruited into the study. Ten participants were excluded from the study altogether, seven on the basis of their haemoglobin phenotype while the others had incompletely filled questionnaires. Overall, the minimum age was 18 years and the maximum 59 years with a mean of 30.48±11.16 years. A total of 102 were females (53.1%) while 90(42.9%) were males. Amongst the cases, 51(53.1%) were females and 45 were males (46.9%) and their mean age was 29.33±10.37 years. The control group was exactly the same in number and gender distribution with the cases (Table 1).

**Table 1:** Age and gender distribution of study participants

Variables	Cases (HbSS)		Controls (HbAA)		X^2^	p-value
**Age (years)**	Frequency	Percentage (%)	Frequency	Percentage (%)		
<20	20	(20.8)	19	(19.8)	1.433	0.153
21-30	42	(43.8)	33	(34.4)		
31-40	21	(21.9)	20	(20.8)		
>40	13	(13.5)	24	(25.0)		
**Gender**						
Males	45	(46.9)	45	(46.9)	0.001	1.000
Females	51	(53.1)	51	(53.1)		

### Prevalence of MTHFR C677T and A1298C mutations

**Figure 1:**
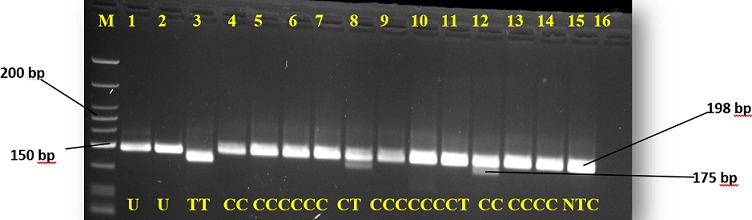
Agarose gel representation of C677T genotyping; M represents the DNA marker (50-700 bp); lanes 1 and 2 are undigested PCR products (198 bp); lane 3 shows TT genotype (175bp); lanes 4-7, 9-11, and 13-15 show the CC genotype (198 bp); lanes 8 and 12 show CT genotype (198 and 175 bp)

**Figure 2:**
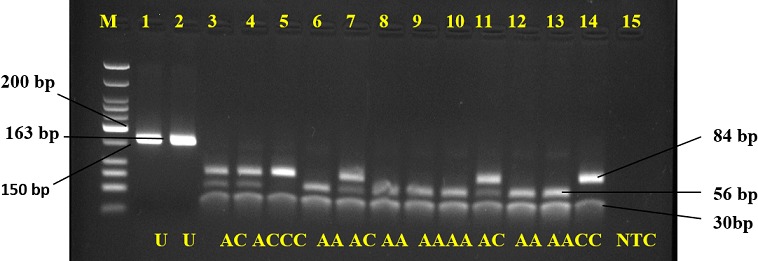
Agarose gel representation of A1298C genotyping; M represents the DNA marker (50-700 bp); lanes 1 and 2 are undigested PCR products (163 bp); lanes 3, 4, 7 and 11 show AC genotype (84, 56, and 30bp); lanes 5 and 14 show the CC genotype (84 and 30 bp); lanes 6, 8-10, 12, and 13 show AA genotype (56 and 30 bp); U = undigested PCR product; NTC = non-template control

The overall prevalence of the C677T mutation among participants was 19.3% (37 of 192), while prevalence of A1298C was 15% (29 of 192) (Figure 1, Figure 2). The genotype frequencies for homozygous dominant (MTHFR 677CC), heterozygous dominant (MTHFR 677CT) and homozygous recessive (MTHFR 677TT) genes among the cases were 83.3%, 15.6% and 1% respectively. In the controls, genotype frequency was 78.1% for the homozygous dominant (CC) and 21.9% for the heterozygous dominant (CT). The homozygous recessive (TT) genotype was not detected among the controls in this study (Table 2). Similarly, the genotype frequencies of the A1298C mutation amongst the cases for the homozygous dominant (AA), heterozygous dominant (AC) and homozygous recessive (CC) were 84.4%, 14.6% and 1% respectively. However, in the controls, genotype frequencies were 85.4% for the homozygous dominant (AA), 12.5% for the heterozygous dominant (AC) and 2.1% for homozygous recessive (CC) genotype (Table 2).

**Table 2:** MTHFR C677T and A1298C genotype frequency distribution for cases and controls

Variant	MTHFR genotype	Cases	Controls	Total	X^2^	p-value
		N (%)	N (%)	N (%)		
C677T	CC	80 (83.3)	75 (78.1)	155 (80.7)	2.160	0.340
	CT	15 (15.6)	21 (21.9)	36 (18.8)		
	TT	1 (1.0)	0 (0.0)	1 (0.5)		
A1298C	AA	81 (84.4)	82 (85.4)	163 (84.9)	1.139	0.570
	AC	14 (14.6)	12 (12.5)	26 (13.5)		
	CC	1 (1.0)	2 (2.1)	3 (1.6)		

Abbreviation: MTHFR = Methylenetetrahydrofolate reductase

### MTHFR C677T and A1298C allelic frequency distribution

The relative frequencies of the 677T alleles for the cases and controls were 8.9% and 10.9% respectively while that of the 1298C alleles was 8.3% in both groups (Table 3).

**Table 3:** MTHFR C677T and A1298C allelic frequency distribution for cases and controls

Variant	Allele	Cases	Controls	X^2^	p-value
		N (%)	N (%)		
C677T	C	175 (91.1)	171 (89.1)	1.103	0.294
	T	17 (8.9)	21 (10.9)		
A1298C	A	176 (91.7)	176 (91.7)	0.033	0.856
	C	16 (8.3)	16 (8.3)		

### Ages and genders of participants with C677T and A1298C mutations

Both mutations in the two groups were not statistically significant when correlated with the ages and genders of the participants except age vs A1298C in controls which was statistically significant (Table 4).

**Table 4:** Association between ages and genders of participants with C677T and A1298C mutations

Participants	Parameters	X^2^	P value
Cases	Age vs C677T	5.12	0.9
	Gender vs C677T	1.15	0.56
	Age vs A1298C	61.54	0.62
	Gender vs A1298C	1.91	0.38
Controls	Age vs C677T	33.68	0.34
	Gender vs C677T	0.01	0.57
	Age vs A1298C	81.22	0.05
	Gender vs A1298C	3.16	0.25

## Discussion

Studies have shown that MTHFR gene mutation results in the diminished activity of the MTHFR enzyme which is crucial in folate metabolism [26, 39]. Polymorphisms of the MTHFR gene in persons with sickle cell anaemia have been suggested as a potential risk for vaso-occlusive events [37, 39]. MTHFR mutations have also been implicated as a probable risk factor for avascular necrosis in sickle cell anaemia [43, 45]. Several genetic factors contribute to the phenotypic expression of individuals with sickle cell anaemia. The MTHFR gene mutations C677T and A1298C are some of such genetic polymorphisms which have been implicated as genetic modifiers of the disease. The prevalence of the C677T mutation among the subjects with HbSS was 16.6% and 21.9% in HbAA controls, showing no statistical difference. Likewise, the prevalence of the A1298C mutation was 15.6% in the cases and 1% lower in the control group. The population frequency of the A1298C allele is less documented than that of the C677T allele and there is inadequately published data about the prevalence of these mutations among Nigerians. These results were higher than a reported prevalence of 5% found in a study of African blacks [51], 9% in another research among African American individuals with sickle cell anaemia [52] and 12% described among Brazilian blacks [51]. However, it is comparable to a study done among a group of African American adults with sickle cell anaemia which reported a prevalence of 15% for the C677T mutation [53]. The prevalence of this mutation has been shown to vary in different populations. A higher prevalence of 36% [51], 38% [54] and 40% [51] has been reported among whites, French-Canadians and Asians respectively. Furthermore, the heterozygous dominant (CT) variant of the MTHFR C677T gene polymorphism was about 34% and 2.2% for the homozygous recessive (TT) variant type in a survey of Brazilians with sickle cell disease [39] and this differs from a frequency of 15.6% for CT variant and 1% for the TT variant found in HbSS individuals in this study. Though not statistically significant, the only homozygous (TT) variant detected was among the HbSS group, which is similar to other studies done in Africa. In a study in sub-Saharan Africa [55], there was no TT genotype identified among 234 individuals tested. In another research [56] involving 89 Africans from four tribes in sub-Saharan Africa, the frequency of the homozygous recessive TT genotype was also not reported. The frequency of the TT genotype among Blacks living outside Africa was also noted to be low [51]. This conflicts with a frequency of TT genotype ranging from 8% among Germans, 18% among Italians and 10-14% among whites living outside Europe to about 21% in Hispanics. These estimates suggest that the homozygous recessive TT variant is less common in Africans than in some other populations. Regarding the A1298C mutation, the homozygous recessive (CC) genotype frequency was 1% in cases and 2.1% in the controls which was not found to be statistically significant. This differs from a CC variant frequency of 6% seen in a survey carried out on 117 healthy Portuguese Caucasians [57]. The 6777T allele frequency ranged from 8.9% in the cases to 10.9% among the controls. This is in keeping with a prevalence of 12% in a study done among Blacks in Brazil [24] and 10% in another one carried out among Blacks in Georgia in the United States of America [24]. However, it was slightly higher than a prevalence of 6.3% generated from a pooled data on Africans in sub-Saharan Africa [24]. It is possible that some environmental or genetic factors not analyzed in this study could have modified the inheritance or expression of the MTHFR gene. Early identification of additional genetic risk factors for either preventive or therapeutic interventions is common clinical practice in the follow up of patients with SCA [30] and contributes to reduced morbidity and mortality in these patients. Even though our study found no significant difference in the prevalence of MTHFR gene mutation among the SCA patients and controls, this may be attributable to a Type II statistical error due to the small sample size used. It is possible that further research using a larger sample size which would be representative of the true prevalence of this mutation would demonstrate a significant difference.

## Conclusion

The prevalence of MTHFR C677T was higher than A1298C mutations among sickle cell anaemia subjects. This research was self-funded, and cost was a major limiting factor. A larger study using more than 96 HbSS and 96 HbAA could be more representative of the aims and objectives of this study.

### What is known about this topic

- MTHFR gene mutation reduces the activity of the MTHFR enzyme and thus leads to hyperhomocysteinaemia;
- This is particularly heightened in patients with sickle cell anaemia who have an increased demand for folate due to their high red cell turnover or shortened red cell lifespan;
- Hyperhomocysteinaemia has been implicated with a number of complications in sickle cell anaemia including avascular necrosis of the femur head, acute chest syndrome etc.

### What this study adds

- The prevalence of MTHFR C677T is higher than A1298C mutations among sickle cell anaemia subjects in Lagos, Nigeria.

## Competing interests

The authors declare no competing interests.
